# Bis(3-carboxy­anilinum) bis­(perchlorate) monohydrate

**DOI:** 10.1107/S1600536809025173

**Published:** 2009-07-04

**Authors:** Lamia Bendjeddou, Aouatef Cherouana, Nasreddine Hadjadj, Slimane Dahaoui, Claude Lecomte

**Affiliations:** a Laboratoire de Chimie Moléculaire, du Contrôle, de l’Environnement et des Mesures Physico-Chimiques, Faculté des Sciences Exactes, Département de Chimie, Université Mentouri de Constantine, 25000 Constantine, Algeria; b Cristallographie, Résonance Magnétique et Modélisation (CRM2), Université Henri Poincaré, Nancy 1, Faculté des Sciences, BP 70239, 54506 Vandoeuvre lès Nancy CEDEX, France

## Abstract

In the structure of the title compound, 2C_7_H_8_NO_2_^+^·2ClO_4_^−^·H_2_O, the ions are connected *via* N—H⋯O, N—H⋯(O,O), O—H⋯O, O—H⋯(O,O) and C—H⋯O hydrogen bonds into a three-dimensional network.

## Related literature

Hydrogen bonds play a crucial role in supra­molecular organization (Jeffrey, 1997 ▶; Nangia & Desiraju, 1998 ▶). Knowledge of hydrogen-bond geometries (Taylor & Kennard, 1984 ▶; Murray-Rust & Glusker, 1984 ▶) and motif formation is vital in the modeling of protein–ligand inter­actions (Tintelnot & Andrews, 1989 ▶; Böhm & Klebe, 1996 ▶). For hydriogen-bond motifs, see: Bernstein *et al.* (1995 ▶). For the structures of organic salts of carboxylic acids, see: Bendjeddou *et al.* (2003 ▶); Cherouana *et al.* (2003 ▶). For a description of the Cambridge Structural Database, see: Allen (2002 ▶);
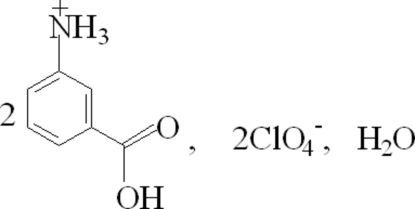


## Experimental

### 

#### Crystal data

2C_7_H_8_NO_2_^+^·2ClO_4_^−^·H_2_O
*M* *_r_* = 493.20
Triclinic, 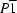

*a* = 4.9170 (3) Å
*b* = 12.4030 (2) Å
*c* = 17.1030 (4) Å
α = 70.520 (2)°
β = 88.697 (3)°
γ = 86.166 (4)°
*V* = 981.13 (7) Å^3^
*Z* = 2
Mo *K*α radiation
μ = 0.41 mm^−1^
*T* = 120 K
0.3 × 0.03 × 0.02 mm

#### Data collection

Enraf–Nonius KappaCCD diffractometer
Absorption correction: none
45183 measured reflections
7071 independent reflections
4775 reflections with *I* > 2σ(*I*)
*R*_int_ = 0.049

#### Refinement

*R*[*F* ^2^ > 2σ(*F* ^2^)] = 0.039
*wR*(*F* ^2^) = 0.102
*S* = 0.99
7071 reflections
292 parameters
H atoms treated by a mixture of independent and constrained refinement
Δρ_max_ = 0.45 e Å^−3^
Δρ_min_ = −0.57 e Å^−3^

### 

Data collection: *CAD-4 Software* (Enraf–Nonius, 1989 ▶); cell refinement: *DENZO* and *SCALEPACK* (Otwinowski & Minor, 1997 ▶); data reduction: *DENZO* and *SCALEPACK*; program(s) used to solve structure: *SIR92* (Altomare *et al.*, 1993 ▶); program(s) used to refine structure: *SHELXL97* (Sheldrick, 2008 ▶); molecular graphics: *ORTEP-3* (Farrugia, 1997 ▶); software used to prepare material for publication: *WinGX* (Farrugia, 1999 ▶), *PARST97* (Nardelli, 1995 ▶), *Mercury* (Macrae *et al.*, 2006 ▶) and *POV-RAY* (Persistence of Vision Team, 2004 ▶).

## Supplementary Material

Crystal structure: contains datablocks global, I. DOI: 10.1107/S1600536809025173/bq2150sup1.cif

Structure factors: contains datablocks I. DOI: 10.1107/S1600536809025173/bq2150Isup2.hkl

Additional supplementary materials:  crystallographic information; 3D view; checkCIF report

## Figures and Tables

**Table 1:** Hydrogen-bonding geometry (Å, °) and unitary motifs

D—H⋯A	D—A	H⋯A	D⋯A	D—H⋯A	Motifs
N1—H1*A*⋯O1*W*^i^	0.89	1.98	2.8664 (18)	171	*D*
N1—H1*B*⋯O6^ii^	0.89	2.11	2.9421 (19)	156	*D*
N1—H1*B*⋯O8	0.89	2.57	3.0343 (18)	114	*D*
N1—H1*C*⋯O1*W*^iii^	0.89	1.98	2.8636 (18)	174	*D*
O1*W*—H1*W*⋯O1^i^	0.840 (14)	2.534 (19)	2.9435 (14)	111.2 (15)	*D*
O1*W*—H1*W*⋯O3^iii^	0.840 (14)	2.258 (14)	3.0356 (16)	153.9 (16)	*D*
O1*W*—H2*W*⋯O1	0.863 (14)	2.019 (15)	2.8504 (17)	161.4 (18)	*D*
N2—H2*A*⋯O6	0.89	2.10	2.9336 (19)	155	*D*
N2—H2*A*⋯O7	0.89	2.57	2.9584 (18)	107	*D*
N2—H2*B*⋯O4^iv^	0.89	2.06	2.9382 (18)	168	*D*
N2—H2*C*⋯O3^v^	0.89	2.55	3.1176 (19)	122	*D*
N2—H2*C*⋯O4^v^	0.89	1.98	2.8683 (18)	175	*D*
O12—H12⋯O11^vi^	0.82	1.82	2.6425 (14)	178	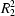 (8)
O22—H22⋯O21^vii^	0.82	1.82	2.6428 (17)	178	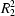 (8)
C13—H13⋯O8	0.93	2.31	3.1193 (19)	145	*D*
C15—H15⋯O2^i^	0.93	2.43	3.3058 (19)	156	*D*
C23—H23⋯O4^iv^	0.93	2.55	3.2529 (19)	133	*D*
C25—H25⋯O8^viii^	0.93	2.49	3.384 (2)	162	*D*
C27—H27⋯O11^vi^	0.93	2.60	3.136 (2)	117	*D*

Symmetry codes: (i) 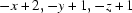; (ii) 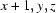; (iii) 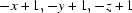; (iv) 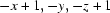; (v) 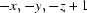; (vi) 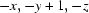; (vii) 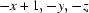; (viii) 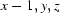. Motifs: *R* = ring; *D* = finite patterns.

**Fig. 1.**
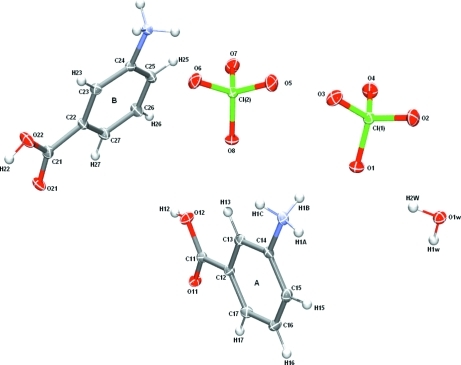
The asymmetric unit of (I), showing the crystallographic numbering scheme. Displacement ellipsoids are drawn at the 50% probability level and H atoms are shown as spheres of arbitrary radii.

**Fig. 2.**
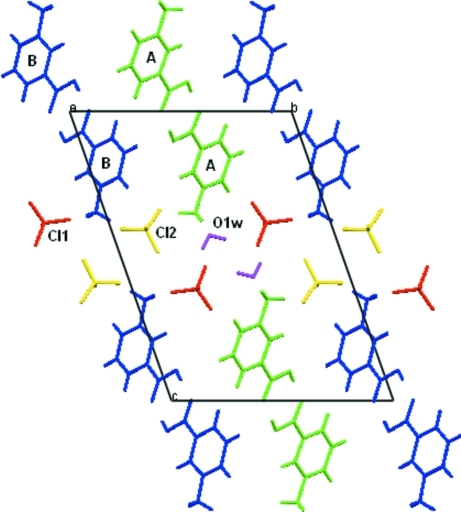
A packing diagram for the title compound, viewed along the a axis, showing the mixture layers.

**Fig. 3.**
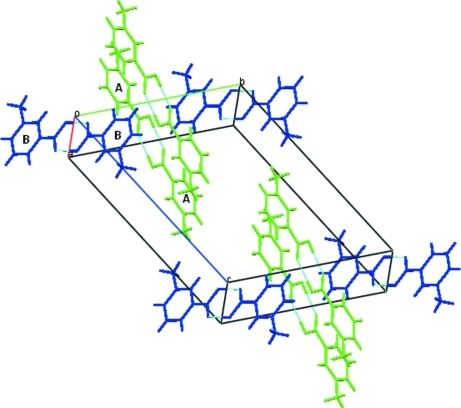
Part of the crystal structure, showing the formation of dimers A and B via O—H···O hydrogen bonds.

**Fig. 4.**
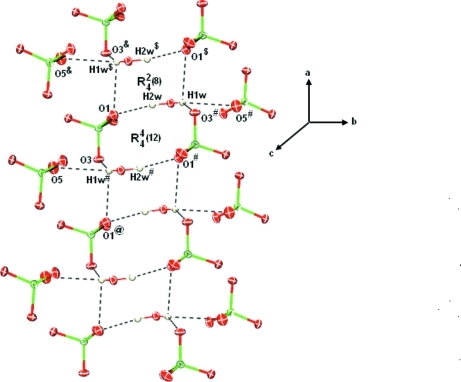
A view of the two-dimensional O—H···O hydrogen-bonded network paralel to the (100) plane of (I), showing the aggregation of R44(12) and R24(8) hydrogen-bonding motifs. Atoms marked with an ampersand (&), an at sign (@), a hash symbol (#) or a dollar sign ($) are at the symmetry positions (1 + x, y, z), (-1 + x, y, z), (1 - x, 1 - y, 1 - z), (2 - x, 1 - y, 1 - z), respectively.

**Fig. 5.**
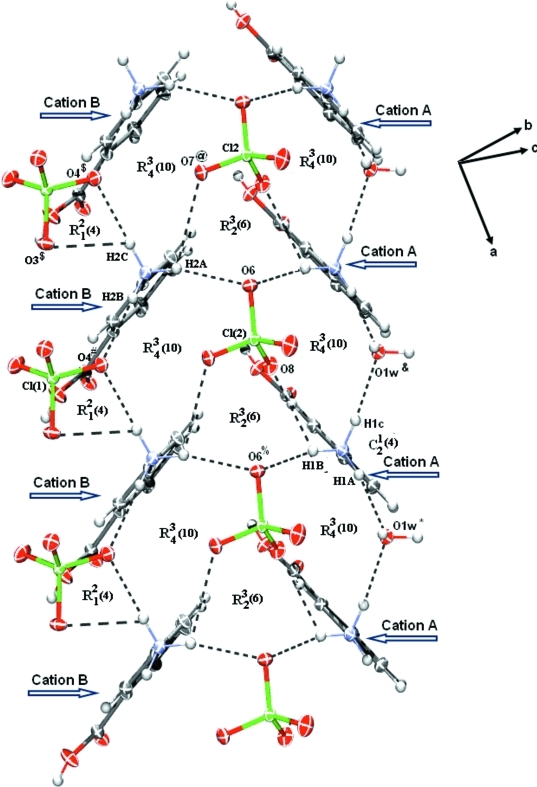
Part of the crystal structure, showing the aggregation of C22(4) R34(10) and R32(6) motifs via N—H···O hydrogen bonds. Atoms marked with an ampersand (&), an at sign (@), a hash symbol (#), dollar sign ($), percent sign (%), or a star sign (*) are at the symmetry positions (1 - x, 1 - y, 1 - z), (-1 + x, y, z), (1 - x, y, 1 - z), (-x, -y, 1 - z), (1 + x, y, z), (2 - x, 1 - y, 1 - z), respectively.

**Fig. 6.**
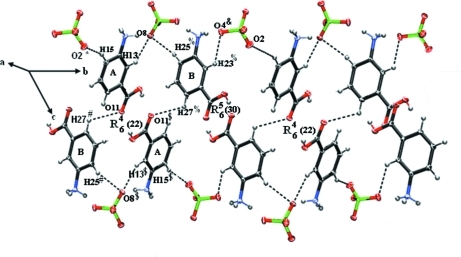
A view of part of the crystal structure of (I), showing the formation of R46(22) and R56(30) rings. Atoms marked with an ampersand (&), a hash symbol (#), dollar sign ($), percent sign (%), or a star sign (*) are at the symmetry positions (2 - x, -y, 1 - z), (-x, 1 - y, -z), (1 - x, 1 - y, -z), (1 + x, y, z), (2 - x, 1 - y, 1 - z), respectively.

## supplementary crystallographic information

### Comment

Hydrogen bonds play a crucial role in supramolecular organization (Jeffrey, 1997; Nangia & Desiraju, 1998). Knowledge of hydrogen-bond geometries (Taylor & Kennard, 1984; Murray-Rust & Glusker, 1984) and motif formation is vital in the modeling of protein-ligand interactions (Tintelnot & Andrews, 1989; Böhm & Klebe, 1996). The supramolecular networks become especially interesting when the cation and anion can participate in hydrogen-bonding. In this regard previous studies have been concerned with organic salts of carboxylic acids (Bendjeddou *et al.*, 2003), Cherouana *et al.*, 2003). The asymmetric unit of (I) (Fig. 1) contains two carboxyanilinium cations (A and B), two perchlorate anion and one water molecule. A proton transfer from the perchloric acid to atom N1 and N2 of *m*-carboxyalinine resulted in the formation of salts.

1- Supramolecular organization:

The structure is formed by double anionic and cationic chains that extend along the *b* axis, giving rise to layers parallel to the plane (b, c). Chains of water molecules are sandwiched between the anionic double chains (Fig2.).

1–1- Overview.

The supramolecular architecture is generated by the nineteen independent interactions of the Table 1. Three types of intermolecular interactions are present in the structure, including O—H···O, N—H···O and C—H···O hydrogen bonds. The presence of water molecule in the structure results in the presence of additional hydrogen bonds. The construction of graphs-set of the twenty hydrogen bonds in this compound has led to a first-level graph set noted: N1 = *R*^2^_2_(8)*R*^2^_2_(8) (Bernstein *et al.*, 1995) (Table 1.).

1–2- The O—H···O hydrogen-bonded network

The carboxylic acid groups at the opposite end of the carboxyanilinium cations forms a centrosymmetric hydrogen-bonded dimers with its counterpart in a cation from an adjacent ribbon and are centered at (0 1/2 0) and (1/2 0 0) respectively for cation A and cation B (Fig. 3). These interactions lead to the graph-set motif *R*^2^_2_(8), which is a characteristic feature found in most salts of 3- and 4-aminobenzoic acid (Cambridge Structural Database; Allen, 2002).

The water molecule, bridges the anionic perchlorate *via the *O—H···O hydrogen bonds. The centrosymmetric hydrogen-bonded rings formed by two water molecules and two Cl(1)O4- anions can be described by the graph-set *R*^4^_4_(12) and *R*^2^_4_(8). The aggregation of this two ring motifs results in an overall one-dimensional hydrogen-bonded chain structure along the [100] direction (Fig. 4).

1–3- The N—H···O hydrogen-bonded network

In the first cationic (A) entity, all ammonium H atoms are involved in hydrogen bonds with the perchlorate (Cl(2) O_4_^-^) anion and water molecule. Two of these interactions link the anions and cations in an alternating fashion into extended chains along the [100] direction, which can be described by the graph-set C^2^_2_(4). The two other interactions are in a crosslink from an adjacent chain C^2^_2_(4). The combination of these two chain motifs generates noncentrosymmetric fused rings which can be described by the graph-set motif *R*^3^_4_(10).

In the second cationic (B) entitie, all ammonium H atoms are involved in hydrogen bonds, with the two different perchlorate ion, so forming an alternating noncentrosymmetric rings a long [100] direction which can be described by the graph-set *R*^3^_4_(10). Only one H atom (H2C) is involved in bifurcated hydrogen bonds with O(3) and O(4) perchlorate atoms, to form a four-membered hydrogen bonded ring *R*^2^_1_(4). The junction betwen this two different cations (A and B) entities are assured by the perchlorate (Cl(2) O_4_^-^) anion *via* N1—H1B···O6, N1—H1B···O8, N2—H2A···O6, N2—H2A···O7 hydrogen bonds, so generete *R*^3^_2_(6) rings along [100] direction (Fig. 5)

1–4- The C—H···O hydrogen-bonded network:

The junction between the cationic entity is consolidated by five weak independent C—H···O hydrogen bonds *via* the perchlorate anions, forming an alternating of *R*^4^_6_(22) and *R*^5^_6_(30)centrosymmetric Rings a long *b* axis (Fig. 6).

### Experimental

*m*-carboxyanilinium acid and perchloric acid were mixed in a 2:2 stoichiometric ratio and dissolved in water. Crystal were obtained by slow evaporation.

### Refinement

H atoms were positioned geometrically and refined in the riding-model approximation, with C—H = 0.97 Å, N—H = 0.89 Å, O—H = 0.82 Å, and with *U*_iso_(H) = 1.2Ueq(C, N) or 1.5Ueq(O). The H atoms of the water molecule were located in a difference Fourier map and reined as riding, with O—H = 0.85 Å and *U*_iso_(H) = 1.5Ueq(O).

### Crystal data

**Table:**

2C_7_H_8_NO_2_^+^·2ClO_4_^−^·H_2_O	*Z* = 2
*M**_r_* = 493.20	*F*(000) = 508
Triclinic, *P*1	*D*_x_ = 1.669 Mg m^−^^3^
Hall symbol: -P 1	Mo *K*α radiation, λ = 0.71073 Å
*a* = 4.9170 (3) Å	Cell parameters from 7071 reflections
*b* = 12.4030 (2) Å	θ = 3.3–32.6°
*c* = 17.1030 (4) Å	µ = 0.41 mm^−^^1^
α = 70.520 (2)°	*T* = 120 K
β = 88.697 (3)°	Needle, brown
γ = 86.166 (4)°	0.3 × 0.03 × 0.02 mm
*V* = 981.13 (7) Å^3^	

### Data collection

**Table:**

Enraf–Nonius KappaCCD diffractometer	4775 reflections with *I* > 2σ(*I*)
Radiation source: fine-focus sealed tube	*R*_int_ = 0.049
graphite	θ_max_ = 32.6°, θ_min_ = 3.3°
ω scans	*h* = 0→7
45183 measured reflections	*k* = −18→18
7071 independent reflections	*l* = −25→25

### Refinement

**Table:**

Refinement on *F*^2^	0 restraints
Least-squares matrix: full	H atoms treated by a mixture of independent and constrained refinement
*R*[*F*^2^ > 2σ(*F*^2^)] = 0.039	*w* = 1/[σ^2^(*F*_o_^2^) + (0.0526*P*)^2^] where *P* = (*F*_o_^2^ + 2*F*_c_^2^)/3
*wR*(*F*^2^) = 0.102	(Δ/σ)_max_ = 0.021
*S* = 0.99	Δρ_max_ = 0.45 e Å^−^^3^
7071 reflections	Δρ_min_ = −0.57 e Å^−^^3^
292 parameters	

### Fractional atomic coordinates and isotropic or equivalent isotropic displacement parameters (Å^2^)

**Table:**

	*x*	*y*	*z*	*U*_iso_*/*U*_eq_	
O11	0.2368 (2)	0.58575 (9)	0.00162 (6)	0.0167 (3)	
O12	0.1451 (2)	0.42214 (9)	0.10223 (6)	0.0176 (3)	
N1	0.6845 (3)	0.40582 (11)	0.35093 (7)	0.0142 (4)	
C11	0.2756 (3)	0.51695 (13)	0.07149 (9)	0.0136 (4)	
C12	0.4736 (3)	0.53492 (13)	0.12947 (9)	0.0131 (4)	
C13	0.4963 (3)	0.45953 (13)	0.21027 (9)	0.0132 (4)	
C14	0.6728 (3)	0.48155 (13)	0.26416 (9)	0.0127 (4)	
C15	0.8297 (3)	0.57585 (13)	0.23929 (9)	0.0154 (4)	
C16	0.8057 (3)	0.65062 (13)	0.15814 (9)	0.0172 (5)	
C17	0.6279 (3)	0.63090 (13)	0.10338 (9)	0.0154 (4)	
O21	0.2424 (2)	0.08889 (10)	−0.00149 (6)	0.0195 (3)	
O22	0.3663 (3)	−0.06863 (10)	0.10513 (7)	0.0231 (4)	
N2	−0.2091 (3)	−0.04135 (11)	0.34838 (7)	0.0132 (3)	
C21	0.2141 (3)	0.02587 (13)	0.07045 (9)	0.0162 (4)	
C22	0.0034 (3)	0.05317 (13)	0.12572 (9)	0.0146 (4)	
C23	−0.0102 (3)	−0.01242 (13)	0.20967 (9)	0.0137 (4)	
C24	−0.1986 (3)	0.02235 (13)	0.25877 (9)	0.0130 (4)	
C25	−0.3762 (3)	0.11787 (14)	0.22733 (9)	0.0171 (4)	
C26	−0.3649 (3)	0.18137 (14)	0.14357 (10)	0.0193 (5)	
C27	−0.1724 (3)	0.14958 (14)	0.09290 (9)	0.0173 (4)	
Cl1	0.58901 (7)	0.29103 (3)	0.61432 (2)	0.0133 (1)	
O1	0.7010 (2)	0.36508 (9)	0.53790 (6)	0.0191 (3)	
O2	0.6783 (2)	0.32216 (11)	0.68248 (7)	0.0237 (4)	
O3	0.2956 (2)	0.30140 (10)	0.60921 (7)	0.0208 (3)	
O4	0.6805 (2)	0.17339 (9)	0.62552 (7)	0.0201 (3)	
Cl2	0.14254 (7)	0.15743 (3)	0.40892 (2)	0.0128 (1)	
O5	0.2017 (3)	0.17301 (10)	0.48568 (7)	0.0244 (4)	
O6	−0.1515 (2)	0.15994 (10)	0.39893 (7)	0.0220 (3)	
O7	0.2591 (2)	0.04883 (9)	0.40697 (7)	0.0207 (3)	
O8	0.2470 (2)	0.24934 (9)	0.34137 (7)	0.0205 (3)	
O1W	0.8260 (2)	0.56613 (10)	0.57195 (7)	0.0156 (3)	
H1A	0.82677	0.42111	0.37583	0.0212*	
H1B	0.70185	0.33315	0.35272	0.0212*	
H1C	0.53190	0.41728	0.37684	0.0212*	
H12	0.02648	0.42104	0.06961	0.0263*	
H13	0.39432	0.39521	0.22790	0.0159*	
H15	0.94884	0.58893	0.27615	0.0185*	
H16	0.90976	0.71430	0.14051	0.0206*	
H17	0.61145	0.68159	0.04935	0.0185*	
H2A	−0.22591	0.00789	0.37622	0.0198*	
H2B	−0.05642	−0.08563	0.36380	0.0198*	
H2C	−0.35139	−0.08481	0.35926	0.0198*	
H22	0.48980	−0.07414	0.07338	0.0345*	
H23	0.10478	−0.07783	0.23186	0.0165*	
H25	−0.50154	0.13923	0.26186	0.0205*	
H26	−0.48533	0.24494	0.12131	0.0232*	
H27	−0.16175	0.19302	0.03700	0.0208*	
H1W	0.826 (4)	0.6178 (12)	0.5253 (8)	0.037 (6)*	
H2W	0.827 (4)	0.5046 (10)	0.5589 (11)	0.039 (6)*	

### Atomic displacement parameters (Å^2^)

**Table:**

	*U*^11^	*U*^22^	*U*^33^	*U*^12^	*U*^13^	*U*^23^
O11	0.0196 (6)	0.0163 (6)	0.0123 (5)	−0.0034 (4)	−0.0031 (4)	−0.0017 (4)
O12	0.0198 (6)	0.0159 (6)	0.0155 (5)	−0.0059 (5)	−0.0060 (4)	−0.0020 (4)
N1	0.0141 (6)	0.0172 (7)	0.0120 (6)	−0.0003 (5)	−0.0017 (5)	−0.0059 (5)
C11	0.0135 (7)	0.0131 (7)	0.0144 (7)	−0.0001 (6)	0.0004 (6)	−0.0049 (6)
C12	0.0130 (7)	0.0140 (7)	0.0124 (7)	−0.0006 (6)	−0.0006 (5)	−0.0044 (6)
C13	0.0129 (7)	0.0123 (7)	0.0148 (7)	−0.0008 (6)	0.0000 (5)	−0.0049 (6)
C14	0.0131 (7)	0.0134 (7)	0.0114 (7)	0.0002 (6)	0.0009 (5)	−0.0040 (5)
C15	0.0144 (7)	0.0173 (8)	0.0165 (7)	−0.0013 (6)	−0.0018 (6)	−0.0080 (6)
C16	0.0173 (8)	0.0144 (8)	0.0200 (8)	−0.0043 (6)	0.0009 (6)	−0.0054 (6)
C17	0.0161 (8)	0.0147 (7)	0.0134 (7)	−0.0008 (6)	−0.0003 (6)	−0.0020 (6)
O21	0.0220 (6)	0.0200 (6)	0.0128 (5)	0.0032 (5)	0.0050 (4)	−0.0019 (4)
O22	0.0253 (7)	0.0218 (6)	0.0164 (6)	0.0091 (5)	0.0070 (5)	−0.0013 (5)
N2	0.0145 (6)	0.0139 (6)	0.0115 (6)	−0.0022 (5)	0.0010 (5)	−0.0044 (5)
C21	0.0172 (8)	0.0159 (8)	0.0155 (7)	0.0011 (6)	0.0004 (6)	−0.0058 (6)
C22	0.0168 (7)	0.0145 (7)	0.0122 (7)	−0.0008 (6)	0.0022 (6)	−0.0043 (6)
C23	0.0138 (7)	0.0128 (7)	0.0130 (7)	0.0003 (6)	−0.0009 (5)	−0.0024 (6)
C24	0.0147 (7)	0.0125 (7)	0.0112 (7)	−0.0032 (6)	0.0006 (5)	−0.0029 (5)
C25	0.0174 (8)	0.0177 (8)	0.0163 (7)	0.0004 (6)	0.0044 (6)	−0.0063 (6)
C26	0.0216 (8)	0.0160 (8)	0.0167 (8)	0.0064 (6)	0.0009 (6)	−0.0020 (6)
C27	0.0214 (8)	0.0157 (8)	0.0119 (7)	0.0015 (6)	0.0021 (6)	−0.0012 (6)
Cl1	0.0122 (2)	0.0142 (2)	0.0143 (2)	−0.0018 (1)	−0.0003 (1)	−0.0057 (1)
O1	0.0220 (6)	0.0172 (6)	0.0156 (5)	−0.0045 (5)	0.0030 (4)	−0.0018 (4)
O2	0.0246 (6)	0.0332 (7)	0.0196 (6)	−0.0101 (5)	−0.0005 (5)	−0.0156 (5)
O3	0.0109 (5)	0.0266 (7)	0.0263 (6)	0.0002 (5)	0.0003 (4)	−0.0109 (5)
O4	0.0175 (6)	0.0120 (5)	0.0289 (6)	0.0014 (4)	−0.0016 (5)	−0.0048 (5)
Cl2	0.0145 (2)	0.0117 (2)	0.0122 (2)	−0.0018 (1)	0.0007 (1)	−0.0039 (1)
O5	0.0355 (7)	0.0256 (7)	0.0138 (5)	−0.0047 (5)	−0.0032 (5)	−0.0081 (5)
O6	0.0127 (5)	0.0219 (6)	0.0340 (7)	−0.0021 (5)	0.0002 (5)	−0.0127 (5)
O7	0.0203 (6)	0.0123 (5)	0.0302 (6)	0.0016 (4)	0.0008 (5)	−0.0087 (5)
O8	0.0284 (7)	0.0152 (6)	0.0155 (5)	−0.0072 (5)	0.0062 (5)	−0.0013 (4)
O1W	0.0175 (6)	0.0136 (5)	0.0145 (5)	−0.0022 (4)	−0.0012 (4)	−0.0028 (4)

### Geometric parameters (Å, °)

**Table:**

Cl1—O2	1.4307 (11)	N1—H1C	0.89
Cl1—O3	1.4418 (11)	C22—C27	1.389 (2)
Cl1—O1	1.4454 (11)	C22—C23	1.397 (2)
Cl1—O4	1.4484 (11)	C22—C21	1.484 (2)
Cl2—O5	1.4282 (11)	C24—C23	1.379 (2)
Cl2—O7	1.4386 (11)	C24—C25	1.383 (2)
Cl2—O8	1.4390 (11)	C14—C13	1.3833 (19)
Cl2—O6	1.4568 (11)	C14—C15	1.385 (2)
O12—C11	1.3208 (17)	C12—C13	1.388 (2)
O12—H12	0.82	C12—C17	1.394 (2)
O1W—H2W	0.863 (9)	C12—C11	1.484 (2)
O1W—H1W	0.840 (9)	C27—C26	1.394 (2)
O11—C11	1.2252 (17)	C27—H27	0.93
O22—C21	1.3135 (19)	C16—C17	1.386 (2)
O22—H22	0.82	C16—C15	1.391 (2)
O21—C21	1.2286 (18)	C16—H16	0.93
N2—C24	1.4732 (18)	C23—H23	0.93
N2—H2A	0.89	C15—H15	0.93
N2—H2B	0.89	C25—C26	1.388 (2)
N2—H2C	0.89	C25—H25	0.93
N1—C14	1.4679 (18)	C17—H17	0.93
N1—H1A	0.89	C13—H13	0.93
N1—H1B	0.89	C26—H26	0.93
			
O2—Cl1—O3	110.41 (7)	C15—C14—N1	119.21 (13)
O2—Cl1—O1	109.66 (7)	C13—C12—C17	120.21 (13)
O3—Cl1—O1	109.24 (7)	C13—C12—C11	120.24 (13)
O2—Cl1—O4	109.82 (7)	C17—C12—C11	119.48 (13)
O3—Cl1—O4	108.57 (7)	O11—C11—O12	123.69 (13)
O1—Cl1—O4	109.11 (7)	O11—C11—C12	122.07 (13)
O5—Cl2—O7	110.85 (7)	O12—C11—C12	114.24 (13)
O5—Cl2—O8	109.49 (7)	O21—C21—O22	123.78 (14)
O7—Cl2—O8	110.20 (7)	O21—C21—C22	121.54 (14)
O5—Cl2—O6	109.62 (7)	O22—C21—C22	114.67 (13)
O7—Cl2—O6	108.29 (7)	C22—C27—C26	120.12 (14)
O8—Cl2—O6	108.34 (7)	C22—C27—H27	119.9
C11—O12—H12	109.5	C26—C27—H27	119.9
H2W—O1W—H1W	102.4 (15)	C17—C16—C15	120.47 (14)
C21—O22—H22	109.5	C17—C16—H16	119.8
C24—N2—H2A	109.5	C15—C16—H16	119.8
C24—N2—H2B	109.5	C24—C23—C22	118.26 (14)
H2A—N2—H2B	109.5	C24—C23—H23	120.9
C24—N2—H2C	109.5	C22—C23—H23	120.9
H2A—N2—H2C	109.5	C14—C15—C16	118.76 (14)
H2B—N2—H2C	109.5	C14—C15—H15	120.6
C14—N1—H1A	109.5	C16—C15—H15	120.6
C14—N1—H1B	109.5	C24—C25—C26	119.11 (14)
H1A—N1—H1B	109.5	C24—C25—H25	120.4
C14—N1—H1C	109.5	C26—C25—H25	120.4
H1A—N1—H1C	109.5	C16—C17—C12	119.84 (14)
H1B—N1—H1C	109.5	C16—C17—H17	120.1
C27—C22—C23	120.41 (14)	C12—C17—H17	120.1
C27—C22—C21	118.25 (13)	C14—C13—C12	118.99 (13)
C23—C22—C21	121.26 (14)	C14—C13—H13	120.5
C23—C24—C25	122.28 (14)	C12—C13—H13	120.5
C23—C24—N2	119.74 (13)	C25—C26—C27	119.79 (15)
C25—C24—N2	117.96 (13)	C25—C26—H26	120.1
C13—C14—C15	121.73 (14)	C27—C26—H26	120.1
C13—C14—N1	119.00 (12)		

### Hydrogen-bond geometry (Å, °)

**Table:**

*D*—H···*A*	*D*—H	H···*A*	*D*···*A*	*D*—H···*A*
N1—H1A···O1W^i^	0.8900	1.9800	2.8664 (18)	171.00
N1—H1B···O6^ii^	0.8900	2.1100	2.9421 (19)	156.00
N1—H1B···O8	0.8900	2.5700	3.0343 (18)	114.00
N1—H1C···O1W^iii^	0.8900	1.9800	2.8636 (18)	174.00
O1W—H1W···O1^i^	0.840 (14)	2.534 (19)	2.9435 (14)	111.2 (15)
O1W—H1W···O3^iii^	0.840 (14)	2.258 (14)	3.0356 (16)	153.9 (16)
N2—H2A···O6	0.8900	2.1000	2.9336 (19)	155.00
N2—H2A···O7	0.8900	2.5700	2.9584 (18)	107.00
N2—H2B···O4^iv^	0.8900	2.0600	2.9382 (18)	168.00
N2—H2C···O3^v^	0.8900	2.5500	3.1176 (19)	122.00
N2—H2C···O4^v^	0.8900	1.9800	2.8683 (18)	175.00
O1W—H2W···O1	0.863 (14)	2.019 (15)	2.8504 (17)	161.4 (18)
O12—H12···O11^vi^	0.8200	1.8200	2.6425 (14)	178.00
O22—H22···O21^vii^	0.8200	1.8200	2.6428 (17)	178.00
C13—H13···O8	0.9300	2.3100	3.1193 (19)	145.00
C15—H15···O2^i^	0.9300	2.4300	3.3058 (19)	156.00
C23—H23···O4^iv^	0.9300	2.5500	3.2529 (19)	133.00
C25—H25···O8^viii^	0.9300	2.4900	3.384 (2)	162.00
C27—H27···O11^vi^	0.9300	2.602	3.136 (2)	117.05

Symmetry codes: (i) −*x*+2, −*y*+1, −*z*+1; (ii) *x*+1, *y*, *z*; (iii) −*x*+1, −*y*+1, −*z*+1; (iv) −*x*+1, −*y*, −*z*+1; (v) −*x*, −*y*, −*z*+1; (vi) −*x*, −*y*+1, −*z*; (vii) −*x*+1, −*y*, −*z*; (viii) *x*−1, *y*, *z*.

### Table 1 Hydrogen-bonding geometry (Å, °) and unitary motifs

**Table:**

D—H···A	D—A	H···A	D···A	D—H···A	Motifs
N1—H1A···O1W^i^	0.89	1.98	2.8664 (18)	171	*D*
N1—H1B···O6^ii^	0.89	2.11	2.9421 (19)	156	*D*
N1—H1B···O8	0.89	2.57	3.0343 (18)	114	*D*
N1—H1C···O1W^iii^	0.89	1.98	2.8636 (18)	174	*D*
O1W—H1W···O1^i^	0.840 (14)	2.534 (19)	2.9435 (14)	111.2 (15)	*D*
O1W—H1W···O3^iii^	0.840 (14)	2.258 (14)	3.0356 (16)	153.9 (16)	*D*
N2—H2A···O6	0.89	2.10	2.9336 (19)	155	*D*
N2—H2A···O7	0.89	2.57	2.9584 (18)	107	*D*
N2—H2B···O4^iv^	0.89	2.06	2.9382 (18)	168	*D*
N2—H2C···O3^v^	0.89	2.55	3.1176 (19)	122	*D*
N2—H2C···O4^v^	0.89	1.98	2.8683 (18)	175	*D*
O1W—H2W···O1	0.863 (14)	2.019 (15)	2.8504 (17)	161.4 (18)	*D*
O12—H12···O11^vi^	0.82	1.82	2.6425 (14)	178	*R*^2^_2_(8)
O22—H22···O21^vii^	0.82	1.82	2.6428 (17)	178	*R*^2^_2_(8)
C13—H13···O8	0.93	2.31	3.1193 (19)	145	*D*
C15—H15···O2^i^	0.93	2.43	3.3058 (19)	156	*D*
C23—H23···O4^iv^	0.93	2.55	3.2529 (19)	133	*D*
C25—H25···O8^viii^	0.93	2.49	3.384 (2)	162	*D*
C27—H27···O11^vi^	0.93	2.60	3.136 (2)	117	*D*

Symmetry codes: (i) -x+2, -y+1, -z+1; (ii) x+1, y, z; (iii) -x+1, -y+1, -z+1; (iv) -x+1, -y, -z+1; (v)  -x, -y, -z+1; (vi) -x, -y+1, -z; (vii) -x+1, -y, -z; (viii)  x-1, y, z.
